# Potential role of SIRT-1 and SIRT-3 as biomarkers for the diagnosis and prognosis of idiopathic pulmonary fibrosis

**DOI:** 10.1186/s12931-024-02796-0

**Published:** 2024-04-27

**Authors:** Fabio Perrotta, Vito D’Agnano, Domenica Francesca Mariniello, Giuseppe Castaldo, Maria Vitale, Mario Cazzola, Andrea Bianco, Filippo Scialò

**Affiliations:** 1 Department of Translational Medical Sciences, University of Campania ‘L. Vanvitelli’, Naples, Italy; 2 U.O.C. Clinica Pneumologica L. Vanvitelli, A.O. dei Colli, Monaldi Hospital, Naples, Italy; 3 CEINGE-Biotecnologie Avanzate Franco Salvatore, Naples, Italy; 4 Department of Molecular Medicine and Medical Biotechnologies, University of Naples “Federico II”, Naples, Italy; 5 Unit of Respiratory Medicine, Department of Experimental Medicine, University of Rome “Tor Vergata”, Rome, Italy

**Keywords:** Idiopathic pulmonary fibrosis, Sirtuins, Biomarkers, Senescence

## Abstract

**Background:**

Idiopathic pulmonary fibrosis (IPF) is a debilitating and progressive lung disease of unknown aetiology, characterized by the relentless deposition of fibrotic tissue. Biomarkers may play a pivotal role as indicators of disease presence, progression, and treatment response. Sirtuins, a family of enzymes with ADP ribosyltransferase or deacetylase activity, have been implicated in several diseases, including pulmonary fibrosis.

**Methods:**

A cross-sectional, prospective, observational single-center study was conducted to investigate the potential role of serum SIRTs levels as biomarkers in patients with IPF. Demographic, clinical, and functional data and serological samples were collected from 34 patients with IPF followed at the Interstital Lung and Rare Diseases Outpatient Clinic of the Vanvitelli Pneumology Clinic, Monaldi Hospital, Naples, Italy and from 19 age-matched controls.

**Results:**

Serum SIRT-1 levels were significantly reduced in IPF patients compared to controls (median IPF 667 [435–858] pg/mL versus controls 925 [794–1173] pg/mL; *p* < 0.001 ). In contrast, serum SIRT-3 levels were significantly increased in IPF patients compared to controls (median IPF 338 [230–500] pg/mL versus controls 154 [99.8–246] pg/mL; *p* < 0.001). There were no statistically significant differences in serum SIRT-6 and SIRT-7 levels between IPF and controls. In addition, we found a significant positive correlation between SIRT-1 and lung function parameters such as FEV_1_% (ϱ=0.417;*p* = 0.016), FVC% (ϱ=0.449;*p* = 0.009) and DL_CO_% (ϱ=0.393;*p* = 0.024), while a significant negative correlation was demonstrated between SIR-1 and GAP score, demonstrating a significant reduction in SIRT-1 in advanced Gender-Age-Physiology (GAP) stages 2–3 compared to GAP stage 1 (*p* = 0.008).

**Conclusions:**

This prospective, cross-sectional study showed that SIRT-1 was associated with lung function and IPF severity and that both SIRT-1 and SIRT-3 could be considered as potential biomarkers of IPF, whereas SIRT-6 and SIRT-7 were not associated with IPF.

## Introduction

Idiopathic interstitial pneumonitis (IIPs) are a subgroup of interstitial lung diseases of unknown etiology characterized by the accumulation of varying degrees of inflammation and fibrosis. Idiopathic pulmonary fibrosis (IPF), the most common subtype of the chronic and progressive IIPs, is associated with significant morbidity and poor prognosis [1, 2]. Both genetic and environmental factors play a key role in the pathogenesis of IPF. Chronic and prolonged environmental exposure and subsequent repetitive micro-lesions of the alveolar epithelium represent the initial events in the development of IPF [3]. Endoplasmic reticulum stress, telomere shortening, mitochondrial dysfunction and epigenetic modifications are some of the alterations that trigger the release of pro-fibrotic mediators and the epithelial-mesenchymal transition leading to myofibroblasts accumulation and fibrosis [3–5]. From a clinical perspective, IPF is characterized by a wide range of phenotypes making its course unpredictable. With the approval of new antifibrotic treatments, the identification of validated biomarkers will be fundamental for clinicians to predict disease prognosis, guide treatment decisions, and provide valuable insights into the disease progression.

**Fig. 1:**
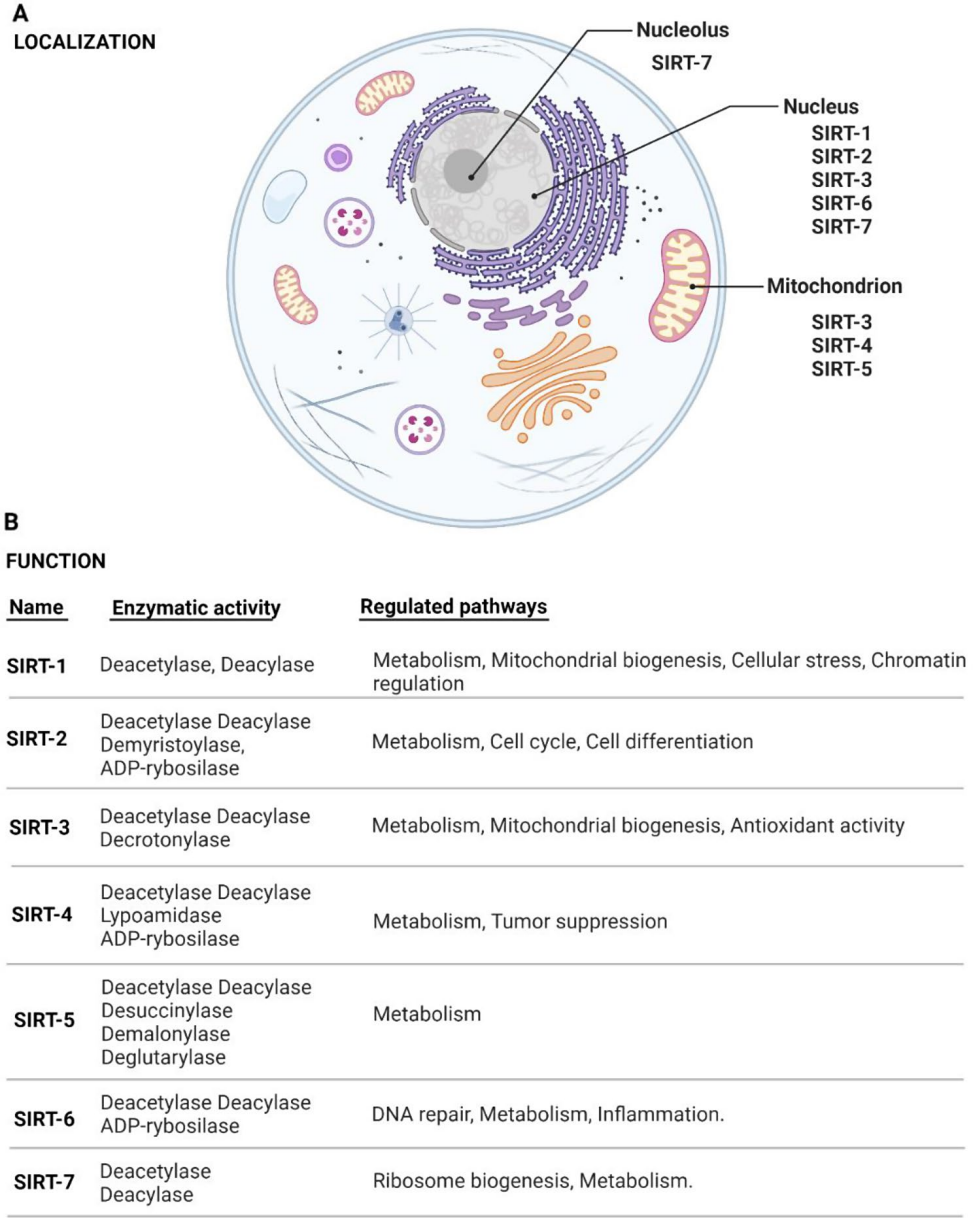
Sirtuins localization (**A**) and function (**B**)

Early evidence suggests that sirtuins (SIRTs), a family of seven members (SIRT-1 to SIRT-7) (Fig. 1) involved in the regulation of cellular metabolism and biological processes such as cell survival, DNA repair, cellular senescence and response to oxidative stress, may play a potential role in fibrotic disorders [6–8]. In addition, SIRTs are known as anti-ageing proteins due to their activity in the processes of DNA repair and maintenance of telomere length [9]. Therefore we conducted a prospective study to investigate the potential role of the serum levels of the key SIRTs as biomarkers in patients with IPF.

## Materials and methods

### Study population

We performed a cross-sectional, prospective, observational single-center study involving IPF patients followed at the Interstital Lung and Rare Diseases Outpatient Clinic of the Vanvitelli Respiratory Diseases Clinic, Monaldi Hospital, Naples, Italy.

Inclusion criteria were: (1) diagnosis of IPF, according to the 2018 ATS/ERS/JRS/ALAT guidelines [10] within 5 years, (2) age ≥ 40 years old, and (3) ability to understand and sign a written informed consent form. Patients were excluded if they had (1) a history of significant exposure to environmental or other known risk factors for pulmonary fibrosis (pro-fibrotic drugs, asbestos, beryllium, radiation, and domestic birds), (2) a clinical diagnosis of any connective tissue disease (CTD) (3) a current diagnosis of asthma or chronic obstructive pulmonary disease (COPD) (4) an acute exacerbation of IPF in the previous 3 months. Demographic and anthropometric characteristics were systematically collected including sex, smoking history, and comorbidities.

Spirometry, body plethysmography and single-breath DL_CO_ were performed in all patients according to the 2022 American Thoracic Society and European Respiratory Society (ATS/ERS) guidelines [11], using Vyntus BODY (Vyaire Medical). The spirometric parameters measured were FEV_1_, FEV1%, FVC, FVC% FEV1/FVC, TLC, TLC%, RV, RV%, DL_CO_, DL_CO_% [12, 13]. Patients also underwent arterial blood gas analysis (ABG) to evaluate the following parameters: pH, partial pressure of oxygen (pO_2_), partial pressure of carbon dioxide (pCO_2_), bicarbonates (HCO3^−^) and lactates. The 6-minute walk test (6MWT) was performed according the ATS guidelines [14].

The Gender-Age-Physiology (GAP) index and GAP stage were calculated for patients with IPF. The GAP index stratifies patients into three stages based on clinical (e.g., sex and age) and physiological (e.g., FVC and DL_CO_) variables. This provides an estimate of 1, 2, and 3-year mortality as previously reported [15]. By adding a measure of exercise capacity (distance walked during the 6MWT and exertional hypoxaemia) to the GAP index, the Distance-Oxygen-Gender-Age-Physiology (DO-GAP) index was obtained [16].

The study was conducted in accordance with the Declaration of Helsinki and received approval from the local Ethics Committee under the reference number 7281. All participants provided written informed consent prior to participation.

### Serum collection

Serum samples were collected from all patients during their visit and allowed to clot for 30 min. They were then centrifuged at 4 °C for 15 min at 1500 rpm. The resulting serum was carefully collected, divided into smaller aliquots and then stored at -80 °C to maintain stability for subsequent analyses.

### Enzyme-linked immunosorbent assay (ELISA)

Serum concentrations of SIRT1 (Assay Gene, catalogue code: HUFI02857), SIRT3 (Assay Gene, catalogue code: HUFI03404), SIRT6 (Assay Gene, catalogue code: HUFI01781) and SIRT7 (Assay Gene, catalogue code: HUFI01031) were measured by ELISA according to manufacturer’s protocol. Briefly, the plate was first washed twice with Wash Buffer 1X (WB) and then loaded with 100 uL of the appropriate standard at different dilutions (1/2, 1/4, 1/8, 1/16, 1/32, 1/64, and the blank) to obtain a standard curve. Serum samples were first diluted, and then 100 uL of each sample were loaded onto the plate in duplicate. The plate was then incubated for 90 min at 37 °C in the dark and washed three times with WB. 100 uL of a solution containing the the appropriate anti-SIRT antibody was added to each well, incubated for 60 min at 37 °C in the dark and washed three times with W. 100 uL of a solution containing HRP-Strepdavidin Conjugate was then added to each well and incubated for 30 min at 37 °C in the dark. After incubation, the plate was washed five times with WB and 90 uL of a solution containing TMB substrate was added to each well and incubated in the dark for 10 min at 37 °C. When the blue color appeared, 50 uL of STOP solution was added to each well, and the absorbance at 450 nm was immediately read using a plate reader.

### Statistical analysis

Categorical or qualitative variables were presented as absolute number and percentage, whereas continuous or quantitative variables were described using either median and inter-quartile range (IQR) or mean and standard deviation (SD), depending on their distribution. The Mann-Whitney test was used to analyze differences in serum levels of sirtuins among different groups. For univariate correlations, Pearson’s or Spearman coefficient were used as appopriate. ROC curves were constructed to assess the diagnostic performance of each sirtuin as a biomarker for IPF. Statistical significance was set at a p-value < 0.05.

## Results

### Evaluation of sirtuins serum levels as biomarkers for diagnosis in IPF

In this study 34 patients diagnosed with IPF and 19 age and gender-matched controls were enrolled (Table 1). The median age in IPF population was 75 years, with 94.1% being current or former smokers. The majority of IPF patients were receiving anti-fibrotic treatments such as nintedanib (62%), pirfenidone (26%) and/or oxygen supplementation (38%). Baseline patients values were used to calculate the GAP index, which was then employed to categorize the IPF population into GAP stage I (35%) and GAP stage II/III (65%) based on severity.

**Table 1:** Demographic, clinical and functional characteristics of the population

Group	IPF (n.34)	Controls (n.19)	p-value
**Age**	75.0 [69-77.1]	71.0 [67–77.0]	0.218
**Gender (Male)**	26(76.3%)	12(63.2%)	0.302
**Smoking**			0.542
Current	9(26.5%)	7(36.8%)	
Former	23(67.6%)	10(52.6%)	
Never	2(5.9%)	2(10.5%)	
**BMI(Kg/m** ^**2**^ **)**	27.0 [25.0-29.8]	25 [25.0–29.0]	0.531
**Comorbidities**			
Systemic Hypertension	19(56%)	.	
Diabetes Mellitus	9(26%)	.	
CAD	6(18%)	.	
Stroke	3(9%)	.	
Congestive Heart Failure	3(9%)	.	
Atrial Fibrillation	2(6%)	.	
Chronic Kidney Failure	4(12%)	.	
GERD	11(32%)	.	
OSA	2(6%)	.	
**Antifibrotic agent**	30(88.2%)	.	
Nintedanib	21(62%)	.	
Pirfenidone	9(26%)	.	
None	4(12%)	.	
**Supplementary long-term oxigen**	13 (38%)		
**GAP index**	4 [3–5]	.	
**GAP Stage**		.	
I	12(35%)	.	
II-III	22(65%)	.	
**DO-GAP Score**	8 [4–14]	.	
**DO-GAP Grade**		.	
1–2	19(55.8%)	.	
3	15 (44.2%)	.	
**FEV**_**1**_ **(L)**	2.10 [1.64–2.36]	.	
**FEV1%**	89.0 [77.0-102]	.	
**FVC (L)**	2.41 [1-96-2.89]	.	
**FVC%**	81.0 [64.0-92.1]	.	
**FEV**_**1**_ **/FVC%**	87.0 [82–92]		
**DL**_**CO**_ **%**	46.9 [32.0-61.3]	.	
**6MWD (mt)**	252 [208–396]	.	
**pH**	7.41 [7.40–7.44]	.	
**pCO**_**2**_ **(mmHg)**	40.0 [37.0–41.0]	.	
**pO**_**2**_ **(mmHg)**	82.0 [72.0-90.5]	.	
**Lactates (mmol/L)**	1.00 [0.82–1.30]	.	
**HCO3** ^**−**^ **(mmol/L)**	25.4 [23.8–26.8]	.	
**SpO**_**2**_ **(%)**	97.2 [96.2–97.9]	.	
**Sirt-1 (pg/mL)**	667[435–858]	925[794–1173]	< 0.001
**Sirt-3 (pg/mL)**	338[230–500]	154[99.8–246]	< 0.001
**Sirt-6 (pg/mL)**	242[146–301]	263[238–328]	0.131
**Sirt-7 (ng/mL)**	4742[3622–6302]	4957[2112–5519]	0.522

Data are presented as median value and interquartile range or as an absolute number (percentage). Coronary Artery Disease(CAD), Gastro-Esophageal Reflux Disease (GERD), Obstructive Sleep Apnea (OSA), Forced Expiratory Volume in 1 s (FEV1), Forced Vital Capacity (FVC), Diffusing capacity of the lungs for carbon monoxide (DLCO)

To enhance the prediction of clinical performance, the GAP index was integrated with measurement of exercise capacity such as the 6MWT and the presence of exertional hypoxaemia to obtain the DO-GAP score. Subsequently, the IPF population was divided into DO-GAP Grade 1–2 (55.8%) and Grade 3 (44.2%).

**Fig. 2:**
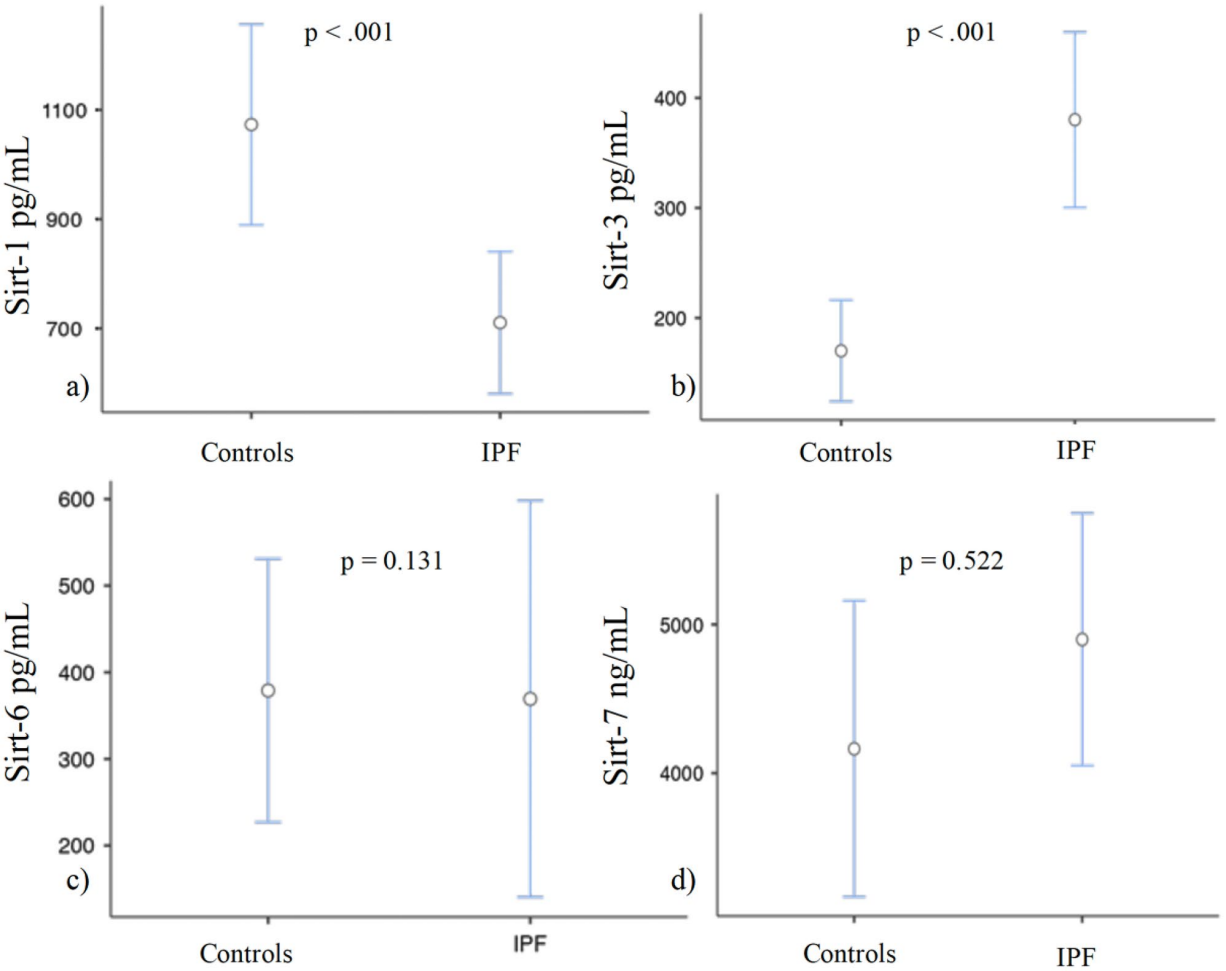
Sirtuins expression in IPF versus controls: Serum levels of SIRT-1 (**A**) SIRT-3 (**B**) SIRT-6 (**C**) and SIRT-7 (**D**) in controls and IPF group

Serum samples to assess the serum concentration of SIRT-1, SIRT-3, SIRT-6 and SIRT-7 were collected from both IPF patients and control subjects at the time of enrolment. As illustrated in Fig. 2a and detailed in Table 1, we observed a significant reduction in serum SIRT-1 levels among IPF patients compared to controls (median IPF 667 [435–858] pg/mL vs. controls 925 [794–1173] pg/mL; *p* < 0.001). Conversely, SIRT-3 serum levels were significantly increased in IPF patients compared to controls (median IPF 338 [230–500] pg/mL vs. controls 154 [99.8–246] pg/mL; *p* < 0.001) (Fig. 2b). No statistically significant differences were observed in serum levels of SIRT-6 and SIRT-7 between IPF and controls. Specifically, as reported in Fig. 2b-c and in Table 1, SIRT-6 levels (median IPF 242 [146–301] pg/mL vs. median controls 263 [238–328] pg/mL; *p* = 0.131) and SIRT-7 levels (4742 [3622–6302] ng/mL vs. controls 4957 [2112–5519] ng/mL; *p* = 0.522) did not exhibit significant variation.

### Sirtuins serum levels association with clinical and physiological disease severity

Given the observed downregulation of SIRT-1 and upregulation of SIRT-3 in the IPF population, we sought to understand whether these changes in enzyme expression correlated with the severity of functional impairment and could have predictive value for disease progression.

**Fig. 3:**
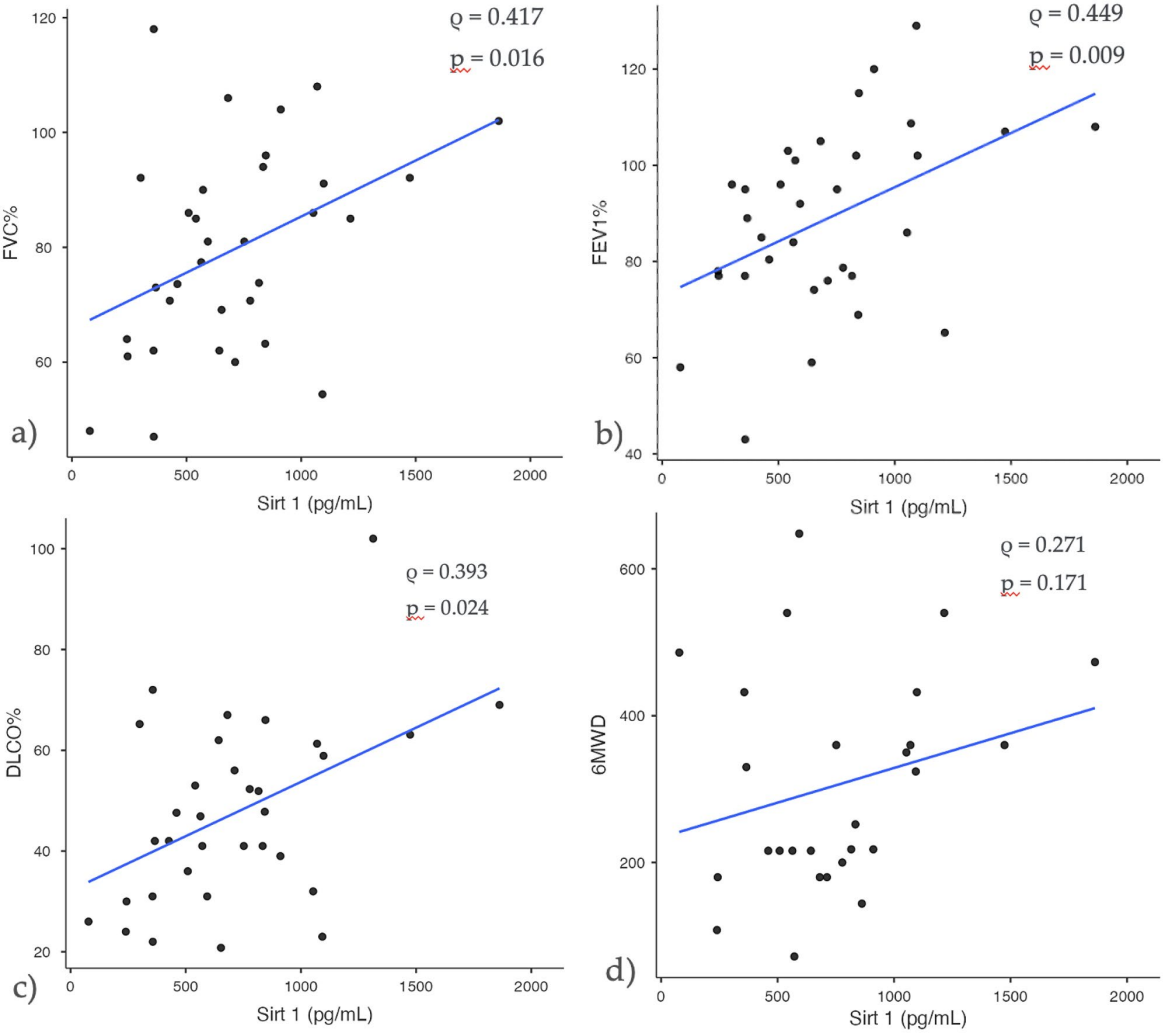
Sirt-1 correlation with main spirometric and functional parameters

**Fig. 4:**
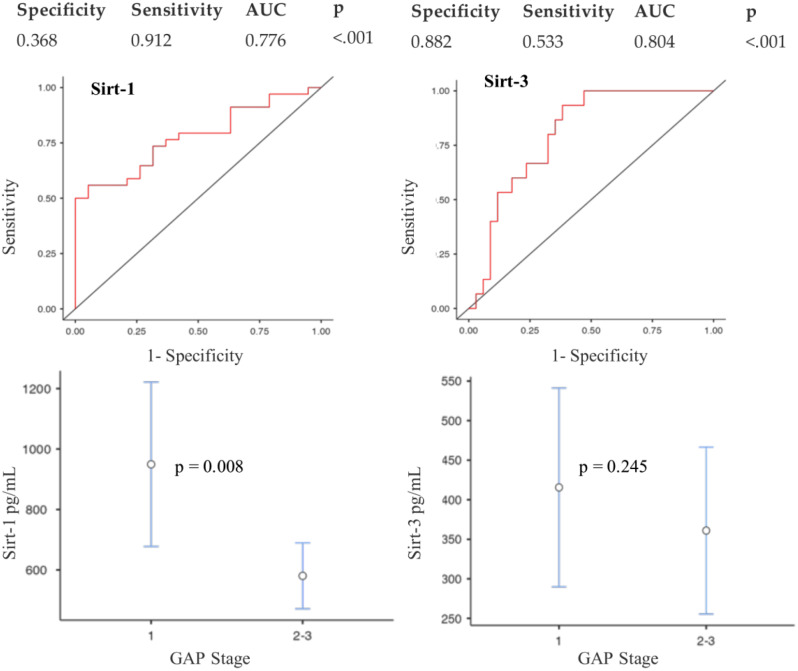
(**a**) Correlation between SIRT-1 and GAP stage; (**b**) Correlation between SIRT-3 and GAP stage; **(c**) ROC curve SIRT-1; (**d**) ROC curve SIRT-3

As shown in Fig. 3 the univariate analysis revealed that SIRT-1 exhibited moderate correlation coefficients with FEV_1_% (ϱ=0.417; *p* = 0.016), FVC% (ϱ=0.449; *p* = 0.009) and DL_CO_% (ϱ=0.393; *p* = 0.024). Conversely, SIRT-3 showed no significant correlation with functional parameters (Suppl. Figure 1). We then examined the association between SIRT-1 and SIRT-3 serum levels and GAP stage (Fig. 4). Notably, SIRT-1 was negatively correlated with GAP stage, demonstrating that SIRT-1 was significantly reduced in advanced GAP stages 2–3 compared to GAP stage 1 (*p* = 0.008) (Fig. 4a). Conversely, SIRT-3 showed no significant correlation with GAP stage (Fig. 4b). To evaluate the diagnostic potential of SIRT-1 and SIRT-3 in discriminating IPF from controls, we constructed ROC curves (Fig. 4c-d).

## Discussion

Sirtuins, a family of deacetylases, play crucial roles in various biological processes including inflammation, metabolism, redox homeostasis, cell proliferation and senescence [17]. In our study of patients with IPF, we observed a reduction in serum levels of SIRT-1 compared to controls and this difference in SIRT-1 expression appeared to correlate with disease severity. Notably, we found that SIRT-1 levels were positively correlated with indicators of lung fuction such as FEV_1_%, FVC% and DL_CO_%, while inversely correlated with GAP stage.

These findings are consistent with those of previous studies in the literature that have also reported decreased levels of SIRT-1 in lung biopsies from patients with pulmonary fibrosis or in lung biopsies from mice treated with bleomycin [18]. As previously described, SIRT-1 exhibits potential anti-fibrotic effects. A study by Deskata et al. in 2022 assessed SIRT-1 levels in plasma and peripheral blood mononuclear cells (PBMC) of IPF patients, and reported significantly lower levels in PBMC from IPF patients compared to controls. Furthermore, the authors concluded that SIRT-1 is predictive of IPF when adjusted for sex and age [19].

Interestingly, induction of SIRT-1 by resveratrol has been shown to ameliorate bleomycin-induced pulmonary fibrosis by inhibiting the recruitment of inflammatory cells, reducing epithelial-mesenchymal transition and TGF-β-mediated extracellular matrix production [20].

In our study population we found significantly higher levels of the mitochondrial protein SIRT-3 in IPF patients compared to controls. However, unlike SIRT-1, SIRT-3 did not correlate with the main spirometric parameters or the GAP stage, indicating a lack of correlation with lung function or disease severity. Nevertheless, ROC analysis showed that SIRT-3 also correlated with the diagnosis of IPF, suggesting its potential as a diagnostic biomarker.

These findings are in contrast with some studies in the existing literature. For example, Cheresh et al. found lower levels of SIRT-3 in alveolar epithelial cells from IPF patients [21]. They proposed two mechanisms by which SIRT-3 exerts its anti-fibrotic effect in mouse models of asbestos-induced pulmonary fibrosis: prevention of mitochondrial DNA damage and subsequent apoptosis of alveolar epithelial cells, as well as inhibition of macrophages recruitment from monocytes into the alveoli following exposure to asbestos Similarly, Rehan et al. found reduced levels of SIRT-3 in lung biopsies from patients with IPF [22]. Moreover, Ji-Hong et al. demonstrated in a mouse model of bleomycin-induced pulmonary fibrosis that baicalein, by increasing SIRT-3 levels, reduced fibroblast senescence by blocking the TGF-β/Smad pathway [23]. In addition, in patients with rheumatoid arthritis, SIRT-3 mRNA expression levels were found to be increased as a compensatory response to oxidative stress and were significantly higher in patients receiving corticosteroid therapy, suggesting that this therapy may influence the levels detected [24]. The conflicting results observed in studies investigating the levels of SIRTs, such as SIRT-1 and SIRT-3, in idiopathic pulmonary fibrosis (IPF) could be attributed to several factors, including differences in the methods used for SIRT assessment and variations in biological samples.

For the other two nuclear proteins, SIRT-6 and SIRT-7, no statistically significant differences were observed between IPF patients and controls in our study. Minagawa et al. performed a study to investigate the role of SIRT-6 in human bronchial epithelial cells [25]. They found that TGF-β-induced cellular senescence correlated with increased levels of p21/waf-1 and IL-1β, a cytokine that induces myofibroblast differentiation. The study suggested that transforming growth factor-β (TGF-β) induces over-expression of SIRT-6 as a compensatory mechanism, which then act on cellular senescence and the IL-1 β production, possibly by regulating the activity of nuclear factor-kB. Conversely, TGF-β did not appear to affect SIRT-1 levels [25].

A study conducted by Wiman et al. showed that SIRT-7 mRNA expression was significantly reduced in fibroblasts from patients with pulmonary fibrosis or systemic sclerosis-associated interstitial lung disease [26]. This study also showed that SIRT-7 levels were impaired in the lungs of bleomycin-treated mice and that transgenic overexpression of SIRT-7 resulted in decreased expression of collagen type I and α-smooth muscle actin [26]. The authors hypothesized that the anti-fibrotic effects of SIRT-7 are mediated through the modulation of Smad3 rather than TGF-β [26].

However, our study has several limitations. Firstly, the cross-sectional study design limits the robustness of our findings as we cannot exclude the potential influence of current antifibrotic therapies on sirtuin expression; only 4 patients in our cohort were antifibrotic-treatment naïve. Furthermore the correlation of sirtuins with clinical, functional, and prognostic indexes would benefit from exploration in larger cohorts with longitudinal assessment of these biomarkers.

Nevertheless, the positive correlation between SIRT-1 levels and lung function/severity of IPF and the increased levels of SIRT-3, along with the lack of significant differences in SIRT-6 and SIRT-7 levels between patients and controls, underscores the potential utility of SIRT-1 and SIRT-3 as potential indicators of disease progression in IPF.

In any case, we believe that there are wider implications of our findings as they may also provide potential avenues for the development of novel therapeutic interventions. In particular, we suggest the potential of targeting specific members of the sirtuin family to address cellular stress, chromatin regulation and mitochondrial dysfunction. This highlights the translational relevance of our results and underscores the importance of further investigation into the role of sirtuins in IPF pathology and treatment.

## Data Availability

All data will made available by the authors upon reasonable request.
